# Correction: Polyunsaturated fatty acid analogues differentially affect cardiac Na_V_, Ca_V_, and K_V_ channels through unique mechanisms

**DOI:** 10.7554/eLife.60141

**Published:** 2020-06-18

**Authors:** Briana M Bohannon, Alicia de la Cruz, Xiaoan Wu, Jessica J Jowais, Marta E Perez, Derek M Dykxhoorn, Sara I Liin, H Peter Larsson

Bohannon BM, de la Cruz A, Xiaoan Wu, Jessica J Jowais, Marta E Perez, Dykxhoorn DM, Sara I Liin, H Peter Larsson. 2020. Polyunsaturated fatty acid analogues differentially affect cardiac Na_V_, Ca_V_, and K_V_ channels through unique mechanisms. *eLife* **9**:e51453. doi: 10.7554/eLife.51453.

Published 24, March 2020

Derek M Dykxhoorn was originally omitted from the authors list. However, he contributed to funding acquisition, provided resources and developed methodology for IPSC-derived cardiomyocyte cultures and we believe he should be included as an author. All authors have agreed to this change.

**New authors list**: Briana M Bohannon, Alicia de la Cruz, Xiaoan Wu, Jessica J Jowais, Marta E Perez, **Derek M Dykxhoorn**, Sara I Liin, H Peter Larsson.

**Original authors list**: Briana M Bohannon, Alicia de la Cruz, Xiaoan Wu, Jessica J Jowais, Marta E Perez, Sara I Liin, H Peter Larsson.

Details for the omitted author:

**Derek M Dykxhoorn**

John P. Hussman Institute for Human Genomics, University of Miami Miller School of Medicine, Miami, United States of America.

**Contribution**: Founding acquisition, Resources, Methodology

**Competing interests**: No competing interests declared

Revised funding statement:

**Funding**

National Institutes of Health (R01-HL131461)

- H Peter Larsson
- Derek M Dykxhoorn

Swedish Research Council (2017-02040)

- Sara I Liin

The funders had no role in study design, data collection and interpretation, or the decision to submit the work for publication.

Original funding statement:

**Funding**

National Institutes of Health (R01-HL131461)

- H Peter Larsson

Swedish Research Council (2017-02040)

- Sara I Liin

The funders had no role in study design, data collection and interpretation, or the decision to submit the work for publication.

The article has been corrected accordingly.

