# Supplementary material for: Polyunsaturated fatty acid analogues differentially affect cardiac NaV, CaV, and KV channels through unique mechanisms
Source: eLife. 2020 Mar 24;9:e51453. doi: 10.7554/eLife.51453 (PMC7159882; doi:10.7554/eLife.51453)
Supplement: Figure 1—source data 1. [file elife-51453-fig1-data1.docx]

| **Effects of lin-taurine on cardiac ion channels** | | | |
| --- | --- | --- | --- |
| **Effects of lin-taurine at 0.2 μM** | | | |
| Channel Name | I/I_0_ (0.2 μM) | ΔV_0.5_ (mV) (0.2 μM) | G_max_/G­_max0_ (0.2 μM) |
| Kv7.1/KCNE1 | 1.0 ± 0.1 | 1.3 ± 0.7 | 1.0 ± 0.03 |
| Cav1.2 | 1.0 ± 0.02 | 0.6 ± 0.5 | 1.0 ± 0.02 |
| Nav1.5 | 1.0 ± 0.01 | -0.3 ± 0.1 | 1.0 ± 0.01 |
| **Effects of lin-taurine at 0.7 μM** | | | |
| Channel Name | I/I_0_ (0.7 μM) | ΔV_0.5_ (mV) (0.7 μM) | G_max_/G­_max0_ (0.7 μM) |
| Kv7.1/KCNE1 | 1.2 ± 0.2 | -0.3 ± 1.4 | 1.1 ± 0.02 |
| Cav1.2 | 0.9 ± 0.1 | 0.9 ± 0.9 | 0.9 ± 0.02 |
| Nav1.5 | 1.0 ± 0.05 | -2.2 ± 0.7 | 1.0 ± 0.01 |
| **Effects of lin-taurine at 2 μM** | | | |
| Channel Name | I/I_0_ (2 μM) | ΔV_0.5_ (mV) (2 μM) | G_max_/G­_max0_ (2 μM) |
| Kv7.1/KCNE1 | 3.2 ± 0.9 | -12.3 ± 3.1 | 1.7 ± 0.4 |
| Cav1.2 | 0.7 ± 0.1 | -0.3 ± 1.8 | 0.7 ± 0.05 |
| Nav1.5 | 0.6 ± 0.07 | -8.8 ± 1.7 | 0.9 ± 0.05 |
| **Effects of lin-taurine at 7 μM** | | | |
| Channel Name | I/I_0_ (7 μM) | ΔV_0.5_ (mV) (7 μM) | G_max_/G­_max0_ (7 μM) |
| Kv7.1/KCNE1 | 7.7 ± 2.9 | -39.9 ± 3.6 | 1.9 ± 0.6 |
| Cav1.2 | 0.6 ± 0.1 | -1.9 ± 2.5 | 0.6 ± 0.05 |
| Nav1.5 | 0.1 ± 0.02 | -23.5 ± 1.9 | 0.5 ± 0.07 |
| **Effects of lin-taurine at 20 μM** | | | |
| Channel Name | I/I_0_ (20 μM) | ΔV_0.5_ (mV) (20 μM) | G_max_/G­_max0_ (20 μM) |
| Kv7.1/KCNE1 | 10.4 ± 4.0 | -73.1 ± 2.6 | 2.0 ± 0.6 |
| Cav1.2 | 0.5 ± 0.1 | -1.2 ± 4.4 | 0.5 ± 0.04 |
| Nav1.5 | 0.1 ± 0.01 | -34.9 ± 1.5 | 0.3 ± 0.04 |
| Table containing source data for the application of the PUFA analogue lin-taurine on the cardiac Kv7.1/KCNE1, Cav1.2, and Nav1.5 channels at every concentration (0.2, 0.7, 2, 7, and 20 μM). Data represented as (mean ± SEM). | | | |
