# Supplementary material for: Polyunsaturated fatty acid analogues differentially affect cardiac NaV, CaV, and KV channels through unique mechanisms
Source: eLife. 2020 Mar 24;9:e51453. doi: 10.7554/eLife.51453 (PMC7159882; doi:10.7554/eLife.51453)
Supplement: Figure 4—source data 2. [file elife-51453-fig4-data2.docx]

| **Effects of pin-taurine on cardiac ion channels** | | | |
| --- | --- | --- | --- |
| **Effects of pin-taurine at 0.2 μM** | | | |
| Channel Name | I/I_0_ (0.2 μM) | ΔV_0.5_ (mV) (0.2 μM) | G_max_/G­_max0_ (0.2 μM) |
| Kv7.1/KCNE1 | 1.1 ± 0.1 | 2.6 ± 2.4 | 1.2 ± 0.1 |
| Cav1.2 | 0.7 ± 0.1 | 2.7 ± 1.9 | 0.9 ± 0.1 |
| Nav1.5 | 1.2 ± 0.1 | 1.6 ± 0.9 | 1.2 ± 0.2 |
| **Effects of pin-taurine at 0.7 μM** | | | |
| Channel Name | I/I_0_ (0.7 μM) | ΔV_0.5_ (mV) (0.7 μM) | G_max_/G­_max0_ (0.7 μM) |
| Kv7.1/KCNE1 | 1.4 ± 0.4 | 2.3 ± 3.5 | 1.3 ± 0.2 |
| Cav1.2 | 0.8 ± 0.1 | 3.9 ± 1.9 | 1.0 ± 0.1 |
| Nav1.5 | 1.2 ± 0.1 | 0.9 ± 1.1 | 1.2 ± 0.2 |
| **Effects of pin-taurine at 2 μM** | | | |
| Channel Name | I/I_0_ (2 μM) | ΔV_0.5_ (mV) (2 μM) | G_max_/G­_max0_ (2 μM) |
| Kv7.1/KCNE1 | 3.0 ± 0.4 | -4.9 ± 3.4 | 2.0 ± 0.4 |
| Cav1.2 | 0.8 ± 0.1 | 3.7 ± 1.8 | 1.0 ± 0.1 |
| Nav1.5 | 1.0 ± 0.1 | -3.0 ± 1.9 | 1.0 ± 0.1 |
| **Effects of pin-taurine at 7 μM** | | | |
| Channel Name | I/I_0_ (7 μM) | ΔV_0.5_ (mV) (7 μM) | G_max_/G­_max0_ (7 μM) |
| Kv7.1/KCNE1 | 6.8 ± 1.0 | -23.8 ± 2.7 | 2.2 ± 0.4 |
| Cav1.2 | 0.7 ± 0.1 | 4.1 ± 2.2 | 0.8 ± 0.1 |
| Nav1.5 | 0.5 ± 0.1 | -16.0 ± 2.7 | 0.4 ± 0.1 |
| **Effects of pin-taurine at 20 μM** | | | |
| Channel Name | I/I_0_ (20 μM) | ΔV_0.5_ (mV) (20 μM) | G_max_/G­_max0_ (20 μM) |
| Kv7.1/KCNE1 | 9.0 ± 1.4 | -51.6 ± 3.5 | 1.9 ± 0.3 |
| Cav1.2 | 0.5 ± 0.1 | 6.0 ± 4.1 | 0.6 ± 0.1 |
| Nav1.5 | 0.2 ± 0.03 | -28.1 ± 2.4 | 0.1 ± 0.02 |
| Table containing source data for the application of the PUFA analogue pin-taurine on the cardiac Kv7.1/KCNE1, Cav1.2, and Nav1.5 channels at every concentration (0.2, 0.7, 2, 7, and 20 μM). Data represented as (mean ± SEM). | | | |
