# Supplementary material for: Polyunsaturated fatty acid analogues differentially affect cardiac NaV, CaV, and KV channels through unique mechanisms
Source: eLife. 2020 Mar 24;9:e51453. doi: 10.7554/eLife.51453 (PMC7159882; doi:10.7554/eLife.51453)
Supplement: Figure 5—source data 2. [file elife-51453-fig5-data2.docx]

| **Effects of pin-glycine on cardiac ion channels** | | | |
| --- | --- | --- | --- |
| **Effects of pin-glycine at 0.2 μM** | | | |
| Channel Name | I/I_0_ (0.2 μM) | ΔV_0.5_ (mV) (0.2 μM) | G_max_/G­_max0_ (0.2 μM) |
| Kv7.1/KCNE1 | 0.7 ± 0.1 | 3.9 ± 0.6 | 1.0 ± 0.1 |
| Cav1.2 | 0.7 ± 0.1 | -3.2 ± 3.0 | 0.9 ± 0.1 |
| Nav1.5 | 1.1 ± 0.05 | 0.4 ± 0.3 | 1.0 ± 0.03 |
| **Effects of pin-glycine at 0.7 μM** | | | |
| Channel Name | I/I_0_ (0.7 μM) | ΔV_0.5_ (mV) (0.7 μM) | G_max_/G­_max0_ (0.7 μM) |
| Kv7.1/KCNE1 | 1.0 ± 0.1 | 2.3 ± 0.2 | 1.2 ± 0.1 |
| Cav1.2 | 0.8 ± 0.1 | -3.3 ± 3.4 | 0.9 ± 0.1 |
| Nav1.5 | 1.1 ± 0.1 | 0.4 ± 0.6 | 1.0 ± 0.03 |
| **Effects of pin-glycine at 2 μM** | | | |
| Channel Name | I/I_0_ (2 μM) | ΔV_0.5_ (mV) (2 μM) | G_max_/G­_max0_ (2 μM) |
| Kv7.1/KCNE1 | 1.1 ± 0.1 | 2.3 ± 0.7 | 1.3 ± 0.1 |
| Cav1.2 | 0.8 ± 0.1 | -4.3 ± 4.1 | 0.9 ± 0.1 |
| Nav1.5 | 1.0 ± 0.1 | -0.3 ± 0.7 | 0.9 ± 0.04 |
| **Effects of pin-glycine at 7 μM** | | | |
| Channel Name | I/I_0_ (7 μM) | ΔV_0.5_ (mV) (7 μM) | G_max_/G­_max0_ (7 μM) |
| Kv7.1/KCNE1 | 2.4 ± 0.2 | -8.5 ± 1.7 | 1.7 ± 0.1 |
| Cav1.2 | 0.8 ± 0.1 | -3.1 ± 4.2 | 0.8 ± 0.2 |
| Nav1.5 | 0.7 ± 0.1 | -4.8 ± 1.9 | 0.7 ± 0.1 |
| **Effects of pin-glycine at 20 μM** | | | |
| Channel Name | I/I_0_ (20 μM) | ΔV_0.5_ (mV) (20 μM) | G_max_/G­_max0_ (20 μM) |
| Kv7.1/KCNE1 | 3.6 ± 0.2 | -19.9 ± 2.4 | 1.7 ± 0.1 |
| Cav1.2 | 0.6 ± 0.2 | -3.4 ± 5.6 | 0.7 ± 0.2 |
| Nav1.5 | 0.3 ± 0.1 | -17.8 ± 4.2 | 0.3 ± 0.1 |
| Table containing source data for the application of the PUFA analogue pin-glycine on the cardiac Kv7.1/KCNE1, Cav1.2, and Nav1.5 channels at every concentration (0.2, 0.7, 2, 7, and 20 μM). Data represented as (mean ± SEM). | | | |
